# Evaluating the Protective Properties of a Xyloglucan-Based Nasal Spray in a Mouse Model of Allergic Rhinitis

**DOI:** 10.3390/ijms221910472

**Published:** 2021-09-28

**Authors:** Marika Lanza, Giovanna Casili, Alessia Filippone, Michela Campolo, Irene Paterniti, Salvatore Cuzzocrea, Emanuela Esposito

**Affiliations:** Department of Chemical, Biological, Pharmaceutical and Environmental Sciences, University of Messina, Viale Ferdinando Stagno D’Alcontres, 31-98166 Messina, Italy; mlanza@unime.it (M.L.); gcasili@unime.it (G.C.); afilippone@unime.it (A.F.); campolom@unime.it (M.C.); ipaterniti@unime.it (I.P.); salvator@unime.it (S.C.)

**Keywords:** allergic rhinitis, nasal barrier permeability, xyloglucan, ovalbumin, cytokines, physical barrier

## Abstract

A breached nasal epithelial barrier plays an important role in driving allergic rhinitis (AR). Corticosteroids remain the standard of care (SoC) but come with side effects, thus alternative safe and effective treatments able to avoid inflammation and restore barrier integrity are needed. The aim of the present study is to evaluate the barrier-forming capacity of a xyloglucan-based nasal spray (XG) and compare its efficacy to several SoC treatments (corticosteroid spray, oral mast-cell stabilizer and oral antihistamine) in reducing allergic responses in addition to its effect when concomitantly administered with an antihistamine. An ovalbumin (OVA)-induced mouse AR model was used. XG shows a significant efficacy in reducing histological damage in AR mice; improves nasal rubbing and histamine-induced hyper-responsiveness. Total and OVA-specific IgE as well as pro-inflammatory cytokines are significantly reduced compared to OVA challenged-mice, with im-proved efficacy when used as an add-on treatment. However, XG reduces mucous secreting cells (PAS-positive) and mucin mRNA expression similar to the corticosteroid-treated mice. XG-spray maintains tight junction protein expression (ZO-1) and conversely decreases HDAC1 significantly; the latter being highly expressed in AR patients. Moreover, the concomitant treatment showed in all of the endpoints a similar efficacy to the corticosteroids. This innovative approach may represent a novel therapeutic strategy for nasal respiratory diseases like AR, reducing undesirable side effects and improving the quality of life in patients.

## 1. Introduction

Allergic rhinitis (AR) and chronic rhinosinusitis (CRS) affect more than 30% of the population worldwide and up to 40% of children, but is commonly undiagnosed [1]. AR is the most common atopic inflammatory disorder of the nasal airways, driven by an allergic immune response to inhaled allergens in sensitized individuals and is characterized by congestion, rhinorrhea (anterior and posterior), sneezing and itching.

Moreover, AR does not only affects the nasal cavity but may also induce headaches, sleep disorders, cognitive dysfunction etc. affecting the quality of life of patients [2].

AR is generally associated with numerous comorbid disorders including asthma, eczema, food allergies and rhinosinusitis. Previous studies have examined the relationship between AR and CRS. Several studies have shown that those with AR are more likely to have sinusitis and vice versa [3].

This disorder is induced by an overreaction of the immune system, specifically an IgE-mediated immune response to an inappropriate stimulus such as dust, pollen, grass, pet dander, mold, etc that trigger a greater inflammatory response [4].

Epithelial cells have an important role as a physical barrier to prevent the entry of pathogens, allergens and other foreign particles [5]. Tight junctions (TJ), which are cell-cell adhesion complexes between epithelial cells, are important for epithelial barrier function [6]. TJ disruption is observed in the epithelial cells of patients with asthma, atopic dermatitis, and nasal allergy [7].

Thus, TJ disruption, namely epithelial barrier dysfunction, is considered involved in the initiation or progression of allergic diseases. This is one of the main reasons why it is important to create a barrier that helps prevent the passage of allergens through the nasal mucosa.

Exposure to these allergens leads to the activation of mast cells, in an IgE-dependent manner, that produce chemical mediators such as histamines and peptide leukotrienes (LTs) [8].

The inflammatory mediators attract, recruit and activate additional inflammatory cells such as eosinophils, neutrophils, and T lymphocytes into the nasal mucosa, determining the late-phase response, which occurs several hours after the first allergen exposure leading to chronic inflammation [9]. The activated T lymphocytes then produce several pro-inflammatory cytokines such as interleukin (IL) IL-4, IL-5, IL-9, IL-13, and GM-CSF that are major components of the inflammatory response in AR as well as allergic asthma. These mediators irritate the sensory nerve endings and blood vessels of the nasal mucosa causing sneezing, watery rhinorrhea, nasal mucosal obstruction and swelling.

The inflammatory cascade is provoked by further activation of mast cells, dendritic cells and T cells. The critical Th2 allergic response is the release of cytokines (eg, IL-3, IL-4, IL-5 and IL-13) that contribute to the progression of inflammatory reactions and promote production of IgE by plasma cells [10].

AR is frequently associated with or precedes asthma, and its impact on asthma (ARIA), endorsed by the WHO, clearly underlines the role of AR as a risk factor for asthma suggesting the need to investigate the bronchial involvement in patients with AR [11].

Indeed, rhinitis has been identified as an independent risk factor for asthma development, and this has allowed us to identify possible strategies for rhinitic patients, with the aim of preventing asthma onset [12].

Nowadays, therapeutic treatments for the management of AR include intranasal corticosteroids (INCS), leukotriene receptor antagonists (LTRAs), antihistamines and mast cell stabilizers. INCS have been chosen as first-line therapy due to their greater anti-inflammatory action in reducing nasal mucosal inflammation, however, its long-term use is often accompanied by common undesirable adverse effects such as a headaches, throat irritation, and nasal dryness, particularly when high doses are used.

Corticosteroids have specific effects on mediators and on the inflammatory cells involved in the allergic process. Mediators that appear to be affected include prostaglandins, leukotrienes, and mast cells [13], and act by inhibiting T lymphocytes, particularly Th2 cells, reducing cytokine production and avoiding eosinophil recruitment.

Antihistamines are effective in relieving early-phase nasal itching, sneezing, rhinorrhea, and ocular symptoms; however, they have not been proven to be effective in managing the late-phase response, particularly congestion, and have no effect on the underlying inflammatory response.

Due to the fact that older first-generation antihistamines are associated with sedation, limiting their use, second-generation nonsedating or less sedating agents are preferred [14]. Other side effects associated to first-generation antihistamines can include psychomotor cognitive impairment, confusion, irritability, and anticholinergic symptoms [15].

Cetirizine, a newer, second-generation antihistamine, blocks the effects of histamine and helps relieve mild to moderate allergy symptoms, but it doesn’t prevent them.

Unlike antihistamines, mast cell stabilizers are effective when used before the allergy challenge occurs. Cromoglycate is the active ingredient in mast-cell stabilizers which is used to prevent the symptoms of asthma. For optimal relief it should be administered regularly for several weeks before allergen exposure. It is relatively safe for most patients, with side effects usually limited to local irritation of the nasal mucosa [16].

Given the adverse side-effects of current pharmacological treatments, the search for alternative treatments has gained increasing interest. Therefore, novel therapeutic agents that are both safe and effective are needed to manage and treat AR and associated conditions.

In this context, natural compounds that protect and restore the mucosal barrier integrity with non-pharmacological action are novel alternatives that can be proposed for the management of different allergy-related diseases, including allergic rhinitis.

In particular, among the natural compounds, great attention is focused on xyloglucan, a plant hemicellulose extracted from the seeds of the tamarind tree (*Tamarindus indica*); thanks to the polysaccharide configuration and mucin-like (i.e., mucin1) characteristics of xyloglucan (XG), it has been defined as a “mucosal protector”. Thanks to its mucoadhesive properties, XG is able to adhere to the mucosa and create a protective mucoadhesive/mechanical film that avoids the attachment and penetration of pathogens and toxins alike, thus restoring the physiological barrier function of the mucosal walls [17].

An in vitro study that used a three-dimensional organotypic airway tissue model (with different cell types) treated with XG, showed that the application of XG to nasal epithelial cells did not impair ciliary movement, enhanced mucociliary clearance and facilitated phagocytosis while reducing mucin secretion, properties which are favorable for the management of rhinitis and associated conditions [18].

The purpose of our study was to evaluate the protective properties of XG in an ovalbumin (OVA)-induced allergic rhinitis on balb/c mice, a model commonly used in studies investigating AR [19,20,21]. Allergens are important to sensitize mice and render them ‘‘allergic’’ with Ovalbumin (OVA) and pollen being the universal reagents for experimental allergy models [22,23,24]. In particular, we studied the protective mucoadhesive properties of XG compared to the standard treatments to mitigate/reduce the nasal allergic responses including related clinical symptoms.

## 2. Results

### 2.1. Xyloglucan Alleviated Symptoms of AR in Mice

**Figure 1:**
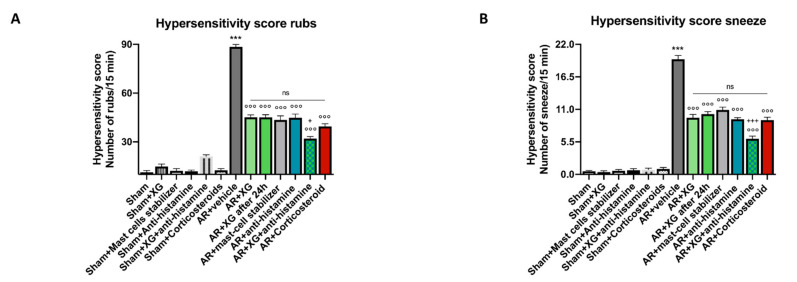
Effects of XG based spray on AR symptoms. A considerable increase in number of rubs and in number of sneezes was detected in AR mice compared to sham groups (**A**,**B**). The administration of XG based spray alone is effective to decrease the number of rubs and sneezing in a manner that is comparable to treatment with the mast-cell stabilizer and antihistamine (**A**,**B**). Moreover, XG based spray in association with antihistamine was able to effectively decrease AR symptoms similarly to corticosteroid-administered animals (**A**,**B**). Data are representative of at least three independent experiments. Values are means ± SEM. One-Way ANOVA test. *** *p* < 0.001 vs. Sham; °°° *p* < 0.001 vs. AR + vehicle; + *p* 0.05 vs. AR + Corticosteroid; +++ *p* 0.001 vs. AR + Corticosteroid. ns: not significant.

Allergic rhinitis (AR) is an atopic disease characterized by symptoms of nasal congestion, clear rhinorrhea, sneezing, postnasal drip, and nasal pruritis [25]. Therefore, the number of sneezes and the amount of nasal rubbing were recorded after the last OVA challenge. As presented in Figure 1, sensitization/challenge of OVA induced significant increases in number of rubs (*p* < 0.001) and in number of sneezes (*p* < 0.001) compared to sham groups. The symptoms score was significantly decreased by the treatments following XG based spray that significantly reduces nasal sneezing symptoms similar to the standard of antihistamine and mast cell stabilizer treatments (*p* < 0.001) instillations. The concomitant treatment with the oral antihistamine significantly reduces nasal rubbing symptoms with similar efficacy if compared to the mice treated with the corticosteroid (*p* < 0.001).

### 2.2. Effect of Xyloglucan on Assessment of Airway Reactivity

**Figure 2:**
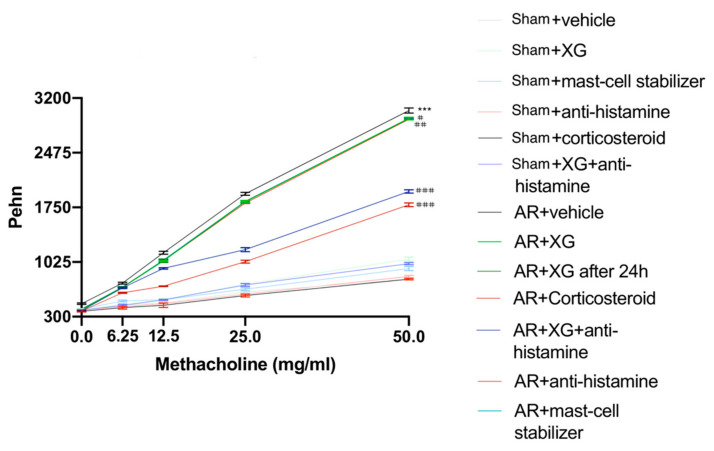
Effect of xyloglucan on assessment of airway reactivity. No considerable variations in Penh were detected in all sham groups following nebulization of saline solution. Contrarily, in AR-mice, the exposure to increasing concentrations of MCh increased Penh levels. On the other hand, such levels were considerably reduced following single treatments with XG, the corticosteroid, antihistamine and the mast cell stabilizer. Additionally, XG and antihistamine co-administration showed an efficacious result comparable to corticosteroid standard care. Data are representative of at least three independent experiments. Values are means ± SEM. Two-Way ANOVA test. *** *p* < 0.001; # *p* < 0.05 vs. AR + vehicle; ## *p* < 0.01 vs. AR + vehicle; ### *p* < 0.001 vs. AR + vehicle.

Enhanced pause (Penh) was used to evaluate changes in pulmonary function and as a method to evaluate airway reactivity, which was determined by using the following equation: Penh = pause × (peak inspiratory boxflow/peak expiratory boxflow). As shown in Figure 2, no changes in Penh were observed following treatment with nebulized normal saline in all experimental groups, while the exposure to increasing concentrations of MCh (6.25 mg/mL and 12.5 mg/mL) increased Penh in AR groups, and was significantly reduced following treatments with XG, corticosteroid, antihistamine and mast cell stabilizer. Particularly, the treatment with XG associated with antihistamine notably reduced the Penh as much as the treatment with corticosteroids (Figure 2). The significant efficacy of XG was also maintained at greater exposures of MCh, almost causing the levels of Penh to reach normal sham values.

### 2.3. Effect of Xyloglucan on Cytokine Quantification in the Nasal Mucosa and BALF of OVA-Sensitized Mice

Pro-inflammatory cytokines (e.g., IL-12, IL-25 and IL-13) play an important role in driving allergic conditions including allergic asthma; IL-33, IL-4 and IL-5 are key factors that drive allergy [26].

**Figure 3:**
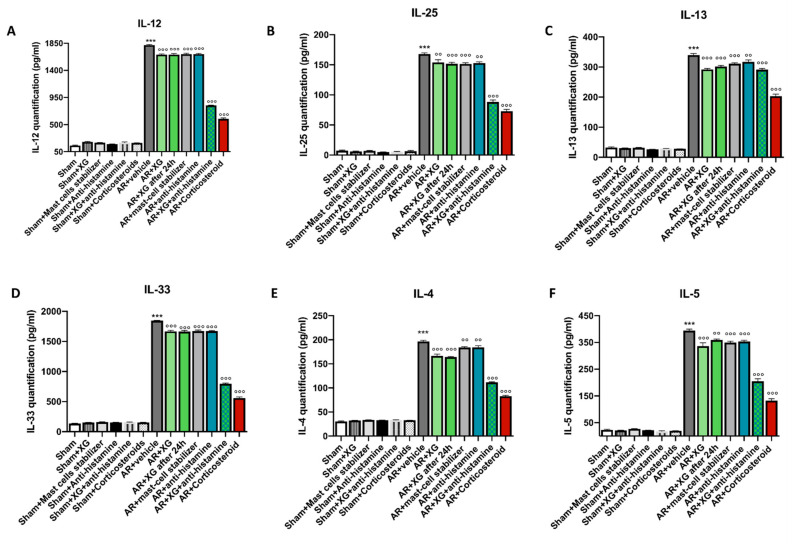
Effect of xyloglucan on cytokine quantification in the nasal mucosa of OVA-sensitized mice. OVA-sensitized mice exposed an extensive upregulation of cytokines in the nasal mucosa tissue, compared to the sham groups (**A**–**F**). However, treatment with XG based spray proved to be successful in reducing the expression of proinflammatory cytokines IL-12, IL-25, IL-13, IL-33, IL-4 and IL-5 in nasal mucosa of mice (**A**–**F**). This decrease was quantitatively greater when XG spray was associated with antihistamine, thus becoming comparable to the effects obtained with corticosteroid administration. Data are representative of at least three independent experiments. Values are means ± SEM. One-Way ANOVA test. *** *p* < 0.001 vs. Sham; °° *p* < 0.01 vs. AR + vehicle; °°° *p* < 0.001 vs. AR + vehicle.

To investigate the effect of XG on pro-inflammatory cytokines levels, we measured the levels of IL-12, IL-25, IL-13, IL-33, IL-4 and IL-5 in the nasal mucosa of OVA-sensitized/challenged mice. Sensitization/challenge of OVA induced a significant decrease in the nasal mucosa levels of all cytokines (Figure 3, *p* < 0.001) compared to sham groups. Treatment with XG, even in mice that were sacrificed 1 day later, as well as anti-histamine and mast cell stabilizer treatments, significantly decreased the nasal mucosa level of IL-12, IL-25, IL-13 and IL-33 (Figure 3A–D respectively *p* < 0.001), with a minor effect on IL-4 and IL-5 (Figure 3E,F, *p* < 0.01 and *p* < 0.05). The treatment with the association of XG plus antihistamine, like the corticosteroid one, significantly decreased the nasal mucosa level of all cytokines (Figure 3A–F) in OVA-sensitized/challenged mice.

**Figure 4:**
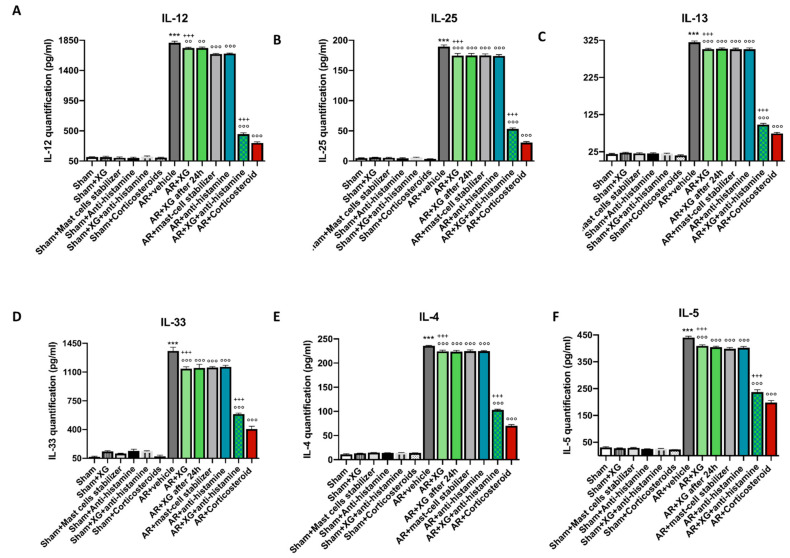
Effect of xyloglucan on cytokine quantification in the BALF of OVA-sensitized mice. OVA-sensitized mice exposed an extensive upregulation of cytokines in the BALF, compared to the sham groups (**A**–**F**). However, treatment with XG-based spray proved to be successful in reducing the expression of proinflammatory cytokines IL-12, IL-25, IL-13, IL-33, IL-4 and IL-5 in BALF of mice (**A**–**F**). This decrease was quantitatively greater when XG spray was associated with antihistamine, thus becoming comparable to the effects obtained with corticosteroid administration. Data are representative of at least three independent experiments. Values are means ± SEM. One-Way ANOVA test. *** *p* < 0.001 vs. Sham; °° *p* < 0.01 vs. AR + vehicle; °°° *p* < 0.001 vs. AR + vehicle; +++ *p* 0.001 vs. AR + Corticosteroid.

Similar results were obtained from the evaluation of cytokines in BALF of OVA-sensitized/challenged mice (Figure 4).

### 2.4. Effect of Xyloglucan on Eosinophil Cell Count into the Nasal Mucosa

Eosinophil infiltration into the nasal mucosa is a major feature of allergic rhinitis [27].

A significant increase in eosinophil cell numbers of the nasal mucosa was observed in OVA-sensitized mice compared to the sham group (*p* < 0.001).

**Figure 5:**
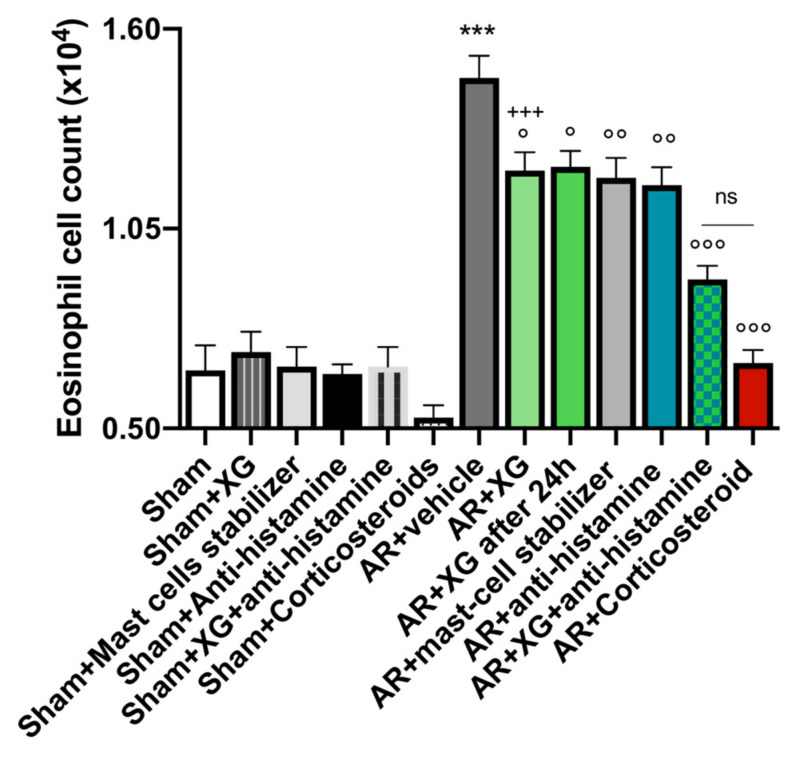
Effect of xyloglucan on eosinophil infiltration in the BALF of OVA-sensitized mice. AR-mice exposed a meaningful proliferation of eosinophil cells in the nasal mucosa, compared to the sham group. Treatment with XG significantly reduced eosinophil cell count comparable to single treatments with antihistamine and mast cell stabilizer. In addition, co-administration of XG based spray and antihistamine as well as corticosteroid treatment similarly reduced eosinophil infiltration. Data are representative of at least three independent experiments. Values are means ± SEM. One-Way ANOVA test. *** *p* < 0.001 vs. Sham; ° *p* < 0.05 vs. AR + vehicle; °° *p* < 0.01 vs. AR + vehicle; °°° *p* < 0.001 vs. AR + vehicle; +++ *p* 0.001 vs. AR + Corticosteroid. ns: not significant.

The treatment with XG, even in mice that were sacrificed one day later, significantly reduced the eosinophils number with similar efficacy to the standard of antihistamine and mast cell stabilizer treatments (Figure 5, *p* < 0.05 and *p* < 0.01). Concomitant treatment with the oral antihistamine displayed comparable efficacy to the corticosteroid-treated group, effectively reducing the eosinophil count (Figure 5, *p* < 0.001).

### 2.5. Effect of XG on Total and OVA-Specific IgE on Serum

**Figure 6:**
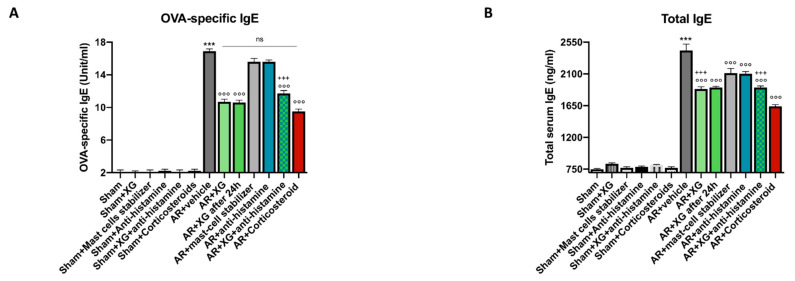
Effect of XG on OVA-specific and total serum IgE levels. Increased levels of IgE were found in the serum of AR-mice compared to the sham animals. The XG-based spray was able to markedly reduce serum total and OVA-specific IgE in a way comparable to mast cell stabilizer and antihistamine single treatments (**A**,**B**). In addition, co-treatment consisting of XG and antihistamine effectively modulated IgE, OVA-specific and total serum levels, similarly to treatment with corticosteroid (**A**,**B**). Data are representative of at least three independent experiments. Values are means ± SEM. One-Way ANOVA test. *** *p* < 0.001 vs. Sham; °°° *p* < 0.001 vs. AR + vehicle; +++ *p* 0.001 vs. AR + Corticosteroid. ns: not significant.

To investigate the effect of XG on IgE production, we measured IgE levels in serum samples of OVA-sensitized/challenged mice using an ELISA assay. Compared with the sham group, mice treated with OVA had significantly higher titers of serum total and OVA-specific IgE (Figure 6). The treatment with XG, even in mice that were sacrificed one day later, significantly reduced total and OVA-specific IgE with a significantly higher efficacy if compared to standard of care treatments, whereas comparable efficacy was observed for total IgE levels (Figure 6A,B, *p* < 0.001). The concomitant treatment of XG with the oral antihistamine was able to reduce OVA-specific and total serum IgE levels with comparable efficacy to corticosteroid-treated mice (Figure 6A,B, *p* < 0. 001).

### 2.6. Histologic Evaluation of Nasal Mucosa following XG Treatment

**Figure 7:**
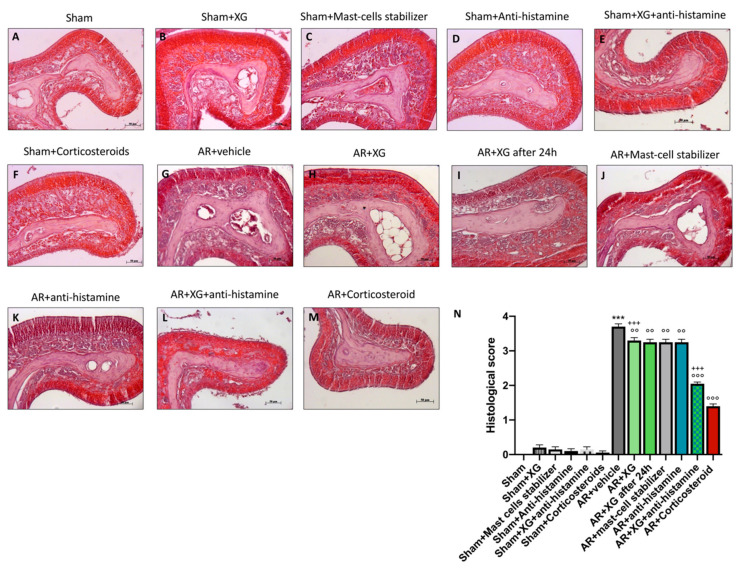
Effect of XG treatment on histological evaluation of nasal mucosa. OVA-challenged nasal mucosa showed edema and tissue inflammation (**G**, histological score **N**) compared to the sham groups (**A**–**F**, histological score **N**). XG-based spray treatment significantly reduced tissue injury (**H**,**I**, histological score **N**); the reduction in OVA-tissue damage was also considerable in single treatments with mast cell stabilizer (**J**, histological score **N**) and antihistamine (**K**, histological score **N**). XG and antihistamine co-administration showed a meaningful protection in histological injury (**L**, histological score **N**) like corticosteroid group (**M**, histological score **N**). Data are representative of at least three independent experiments. Values are means ± SEM. One-Way ANOVA test. *** *p* < 0.001 vs. Sham; °° *p* < 0.01 vs. AR + vehicle; °°° *p* < 0.001 vs. AR + vehicle; +++ *p* 0.001 vs. AR + Corticosteroid.

Nasal tissue was isolated from each mouse and stained with hematoxylin and eosin (H&E). The severity of inflammation, the thickness of the olfactory mucosa and olfactory bulb glomerulus were measured by microscopical analysis, as shown in Figure 7. Sensitization/challenge of OVA in AR groups resulted in histopathological changes such as epithelial disruption, edema of the olfactory mucosa, hypertrophy of the olfactory bulb glomerulus and hyperplasia of goblet cells (Figure 7G, histological score 7N), compared to sham groups (Figure 7A–F, histological score 7N).

Treatments with XG significantly reduces histological damage in AR mice, decreasing hyperplasia of goblet cells and olfactory mucosa thickness (Figure 7H,I, histological score 7N), comparable to the mast-cell stabilizer and antihistamine treatments (Figure 7J,K respectively, histological score 7N). The treatment with the association of XG plus antihistamine significantly improved the histological damage due to OVA-sensitization, reducing the hyperplasia of goblet cells and the thickness of the olfactory mucosa (Figure 7L, histological score 7N), comparable with corticosteroid-treated mice (*p* < 0.001; Figure 7M, histological score 7N).

### 2.7. Effect of XG on Permeability of the Nasal Mucosa (Tight Junctions)

**Figure 8:**
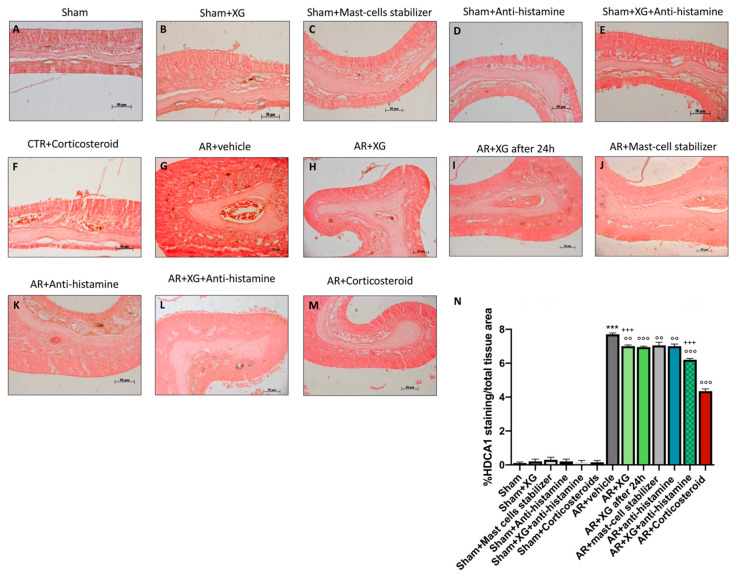
Effect of XG treatment on HDAC1 expression. OVA-challenged nasal mucosa exposed an evident increase of HDAC1 expression (**G**, histological score **N**) compared to the sham groups (**A**–**F**, histological score **N**); Single treatments with XG-based spray significantly reduced HDAC1 expression (**H**,**I**, histological score **N**) similarly to antihistamine (**K**, histological score **N**) and mast cell stabilizer (**J**, histological score **N**) administrations. Co-treatment consisting of XG and antihistamine proved to be efficacious in decrease HDAC1 levels (**L**, histological score **N**), analogously to treatment with corticosteroid (**M**, histological score **N**). Data are representative of at least three independent experiments. Values are means ± SEM. One-Way ANOVA test. *** *p* < 0.001 vs. Sham; °° *p* < 0.01 vs. AR + vehicle; °°° *p* < 0.001 vs. AR + vehicle; +++ *p* 0.001 vs. AR + Corticosteroid.

Histone deacetylase (HDAC) activity has been identified as a crucial driver of allergic inflammation and tight junction dysfunction [7]. Histone deacetylases (HDACs) and Zonula Occludens (ZO)-1 expressions were performed in nasal mucosa by immunostaining. A positive staining for HDAC1 was observed in the AR group (Figure 8G, histological score 8N) compared to sham animals (*p* < 0.001; Figure 8A–F, histological score 8N). A significant reduction in HDAC1-positive cells was obtained in AR groups treated with XG (Figure 8H,I, histological score 8N) or with antihistamine (Figure 8K, histological score 8N) or with mast cell stabilizer (Figure 8J, histological score 8N). The treatment with XG plus antihistamine (Figure 8L, histological score 8N), as well as the treatment with corticosteroid (Figure 8M, histological score 8N), notably reduced the positive staining for HDAC1 (*p* < 0.001).

**Figure 9:**
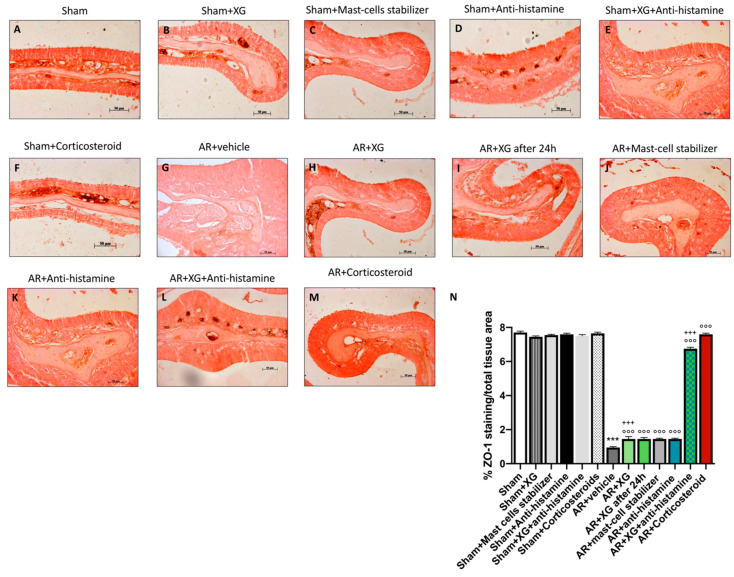
Effect of XG treatment on ZO-1 expression. OVA-sensitized nasal mucosa revealed a marked reduction of ZO-1 expression (**G**, histological score **N**) compared to the sham animals (**A**–**F**, histological score **N**); XG-based spray treatment significantly preserved ZO-1 expression (**H**,**I**, histological score **N**) as well antihistamine (**K**, histological score **N**) and mast cell stabilizer (**J**, histological score **N**) administrations. However, a quantitatively more substantial effect was obtained from the co-administration of XG and antihistamine (**L**, histological score **N**) and corticosteroid treatment (**M**, histological score **N**). Data are representative of at least three independent experiments. Values are means ± SEM. One-Way ANOVA test. *** *p* < 0.001 vs. Sham; °°° *p* < 0.001 vs. AR + vehicle; +++ *p* 0.001 vs. AR + Corticosteroid.

**Figure 10:**
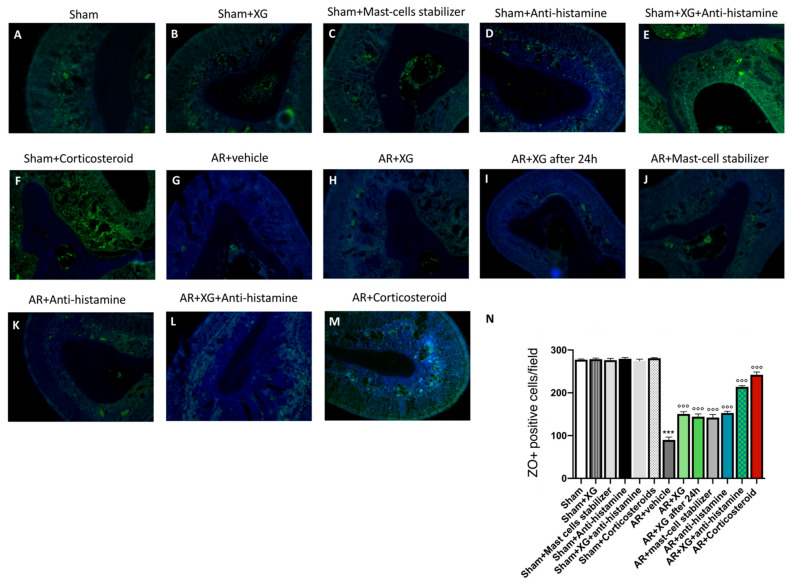
Effect of XG treatment on ZO-1 expression. OVA-challenged nasal mucosa exposed a evident decrease of ZO-1 expression (**G**, histological score **N**) compared to the sham animals (**A**–**F**, histological score **N**); XG-based spray treatment meaningfully preserved ZO-1 expression (**H**,**I**, histological score **N**) as well antihistamine (**K**, histological score **N**) and mast cell stabilizer (**J**, histological score **N**) treatments. A quantitatively more substantial effect was obtained from the co-administration of XG and antihistamine (**L**, histological score **N**) as well as corticosteroid administration (**M**, histological score **N**). Data are representative of at least three independent experiments. Values are means ± SEM. One-Way ANOVA test. *** *p* < 0.001 vs. Sham; °°° *p* < 0.001 vs. AR + vehicle.

Conversely, the positive staining for ZO-1 was significantly reduced in the AR group (Figure 9G, histological score 9N), compared to sham mice (*p* < 0.001; Figure 9A, histological score 9N). The treatments (Figure 9H,I, histological score 9N) and the association of XG plus antihistamine (Figure 9L, histological score 9N) significantly restored the ZO-1 positive cells in nasal mucosa (*p* < 0.001). These results were confirmed by immunofluorescence staining (Figure 10).

### 2.8. Effect of XG on Mucous Secretion

**Figure 11:**
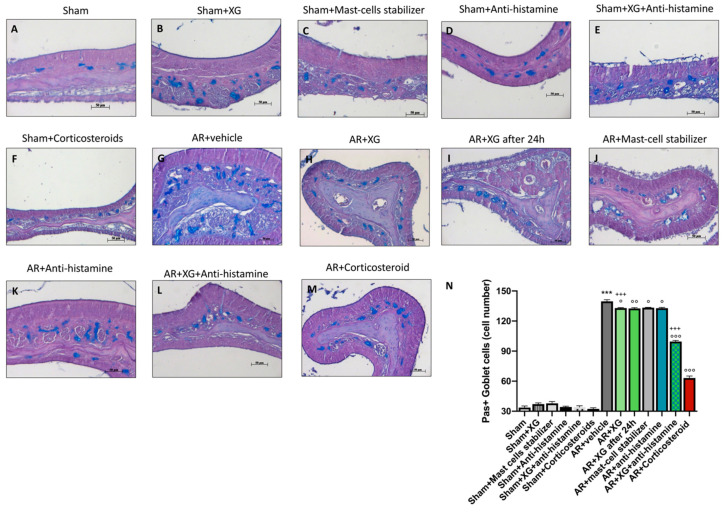
Effect of XG treatment on PAS staining. An important increase of PAS positive cells was detected in AR-mice (**G**, histological score **N**), compared to sham mice (**A**–**F**, histological score **N**). XG treatment (**H**,**I**, histological score **N**) diminished the mucous secretion observed as PAS-positive cell number with equivalent effectiveness to the antihistamine (**K**, histological score **N**) and mast-cell stabilizer (**J**, histological score **N**) treatments. In addition, co-treatment with XG and antihistamine (**L**, histological score **N**) reduced PAS-positive cells, in a comparable way with corticosteroid treatment (**M**, histological score **N**). Data are representative of at least three independent experiments. Values are means ± SEM. One-Way ANOVA test. *** *p* < 0.001 vs. Sham; ° *p* < 0.05 vs. AR + vehicle; °° *p* < 0.01 vs. AR + vehicle; °°° *p* < 0.001 vs. AR + vehicle; +++ *p* 0.001 vs. AR + Corticosteroid.

Goblet cell hyperplasia and metaplasia are common features of chronic asthmatic airways, therefore the degree of mucous secretion was assessed with a Periodic acid-Schiff (PAS) staining, performed on nasal tissue sections. PAS staining was used to evaluate mucus production in epithelia [28]. Interestingly, a significant increase of PAS positive cells, indicated as blue spots, was observed in AR group (Figure 11G, histological score 11N), compared to sham groups (*p* < 0.001; Figure 11A–F, histological score 11N). The treatment with XG (Figure 11H,I, histological score 11N) reduced the mucous secretion observed as PAS-positive cell number with comparable efficacy to the antihistamine (Figure 11K, histological score 11N) and mast-cell stabilizer (Figure 11J, histological score 11N) treatments (*p* < 0.01).

The association of XG with antihistamine significantly reduced the number of PAS-positive cells (Figure 11L, histological score 11N), comparable with corticosteroid treatment (*p* < 0.001; Figure 11M, histological score 11N).

**Figure 12:**
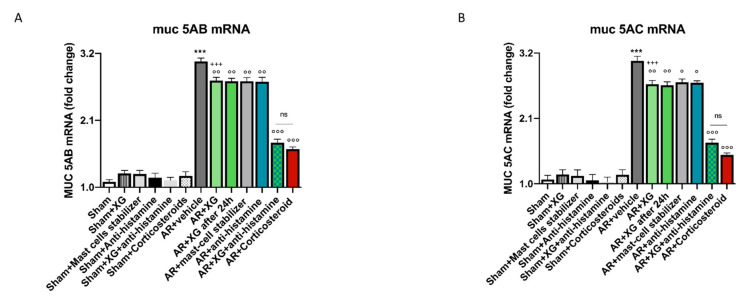
Effect of XG treatment on MUC5AB and MUC5AC expressions. MUC5AB and MUC5AC expression were increased in AR-mice compared to the sham group (**A**,**B**). XG single treatments considerably reduced the mRNA expression for both mucins with similar efficacy to the to the antihistamine and mast-cell stabilizer treatments (**A**,**B**). Moreover, XG and antihistamine co-treatment has been shown to decrease MUC5AB and MUC5AC expression, analogously to treatment with corticosteroid (**A**,**B**). Data are representative of at least three independent experiments. Values are means ± SEM. One-Way ANOVA test. *** *p* < 0.001 vs. Sham; ° *p* < 0.05 vs. AR + vehicle; °° *p* < 0.01 vs. AR + vehicle; °°° *p* < 0.001 vs. AR+vehicle; +++ *p* 0.001 vs. AR+Corticosteroid. ns: not significant.

We also studied altered MUC5AC and MUC5B gene expression consistently observed in rhinitis models, therefore, mucins expression in sinus mucosa was analyzed using RT-qPCR analysis, in particular MUC5AB and MUC5AC expression. A significant increase in both mucins mRNA expression levels was observed in AR groups compared to sham groups (*p* < 0.001; Figure 12A,B). The treatment with XG significantly reduced the mRNA expression for both mucins with similar efficacy to the standard of care treatments (*p* < 0.001), indicating improved allergy response (*p* < 0.01; Figure 12A,B). The treatment with the association of XG plus antihistamine notably reduced the mRNA expression of both mucins compared to treatment with corticosteroid (*p* < 0.001; Figure 12A,B).

### 2.9. Effect of XG on Eosinophils Infiltration

**Figure 13:**
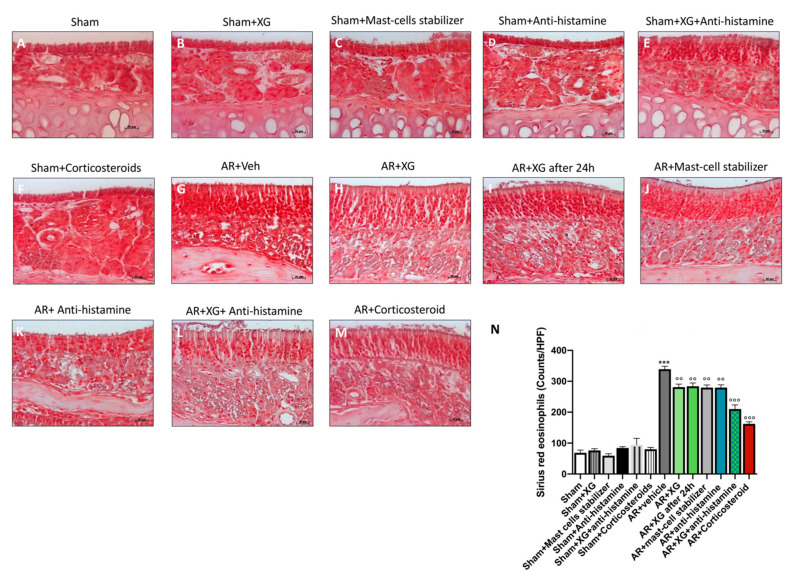
Effect of XG on eosinophils infiltration. Eosinophils count was increased in AR-mice (**G**) compared to the sham groups (**A**–**F**, histological score **N**). XG single treatments (**H**,**I**, histological score **N**) considerably reduced eosinophils infiltration with comparable efficacy to the to the antihistamine (**K**, histological score **N**) and mast-cell stabilizer (**J**, histological score **N**) treatments. Moreover, XG and antihistamine co-administration (**L**, histological score **N**) proved to decrease eosinophils infiltration, analogously to corticosteroid treatment (**M**, histological score **N**). Data are representative of at least three independent experiments. Values are means ± SEM. One-Way ANOVA test. *** *p* < 0.001 vs. Sham; °° *p* < 0.01 vs. AR + vehicle; °°° *p* < 0.001 vs. AR + vehicle.

Eosinophils infiltration in nasal tissue was evaluated by Sirius red staining. An increased number of eosinophils was detected in the AR mice group (Figure 13G, histological score 13N, compared to sham groups (Figure 13A–F, histological score 13N).

However, XG administration significantly reduced eosinophils infiltration after OVA sensitization (Figure 13H,I, histological score 12N), in a comparable way to the mast-cell stabilizer and antihistamine treatments (Figure 13J,K respectively, histological score 13N). In addition, also XG plus antihistamine co-treatment meaningfully decreased eosinophils count (Figure 13L, histological score 13N) in an equally significant way to treatment with corticosteroids (*p* < 0.001; Figure 13M, histological score 13N).

### 2.10. Effect of XG on Cell Viability

**Figure 14:**
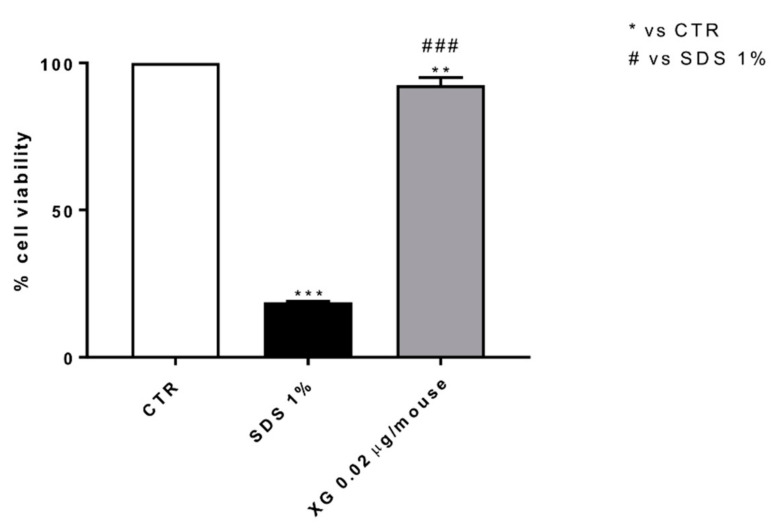
Effect of XG treatment on L-929 cell viability. MTT assay revealed that the treatment with XG at the concentration of 0.02 μg didn’t reduce L-929 cell viability. Data are representative of at least three independent experiments. Values are means ± SEM. One-Way ANOVA test. ** *p* < 0.01 vs. control (CTR); *** *p* < 0.001 vs. CTR; ### *p* < 0.001 vs. SDS 1%.

To evaluate the cytotoxic effect of XG, we conducted a MTT assay on Murine Fibroblast L-929 cells. Our data revealed that the treatment with XG at the concentration of 0.02 μg preserved cell viability as shown in Figure 14.

## 3. Discussion

AR represent an IgE-mediated inflammatory event which involve the nasal airways. Airborne allergens stimulate inflammatory cell penetration within the nasal mucosa, including basophils, eosinophils and mast cells that release several mediators, like histamine, cysteinyl-leukotrienes and prostaglandins, which drive the inflammatory response. AR includes a period of sensitization starting with allergen exposure leading up to inflammation and clinical manifestations of an allergic reaction [29].

AR prevalence has increased exponentially over the past few decades, especially in developed and industrialized countries. However, despite the identification of factors concurrent to the etiology of AR, such as precipitating factors and predisposing factors, the etiopathogenesis of the disease is not yet fully understood. Relatedly, several putative factors have been proposed in particular: lifestyle; exposure to allergens, pollution, and irritants (i.e., smoke, gas, etc.); dietary changes, infections and stress [11]. Thus, the cooperation among environmental factors and individual susceptibility is crucial.

Currently, the therapeutic options available for AR treatment are effective in managing symptoms by reducing the inflammatory process and dampening the immune response; however, they are accompanied by adverse side effects [30].

Whereby, there is an increasing need to treat AR with natural compounds that are safe and effective, able to reduce the adverse side effects.

In particular, a novel approach to create a barrier that protects mucosa from allergens is gaining increasing interest. In this regard, XG, a neutral polysaccharide possessing mucomimetic and mucoadhesive properties, is known for its barrier functionality, thus decreasing bacterial adherence or invasion, preserving tight junctions and paracellular flux, as demonstrated by several in vitro and in vivo findings [31,32,33].

Therefore, the function of mucosal-barrier compounds is attracting increasing attention among scientists as an innovative way to prevent various diseases, including respiratory disorders [32,34].

Thus, in the present study we evaluated the capacity of a nasal spray containing XG to form a protective layer over the sinonasal mucosa in order to assess the reduction of the inflammatory cascade in an in vivo model of ARinduced by OVA sensitization.

Notably, after one week of treatment, we observed that the XG-based spray reduced symptoms of rhinorrhea and itching compared to a physiological saline nasal spray, with comparable efficacy also seen with other mainstream treatments such as antihistamines and mast-cell stabilizers.

The Penh test is a very useful instrument to analyze the immediate-type hypersensitivity response in the nasal airway. Variations in Penh during the progress of nasal allergy are effective correlates with many markers of nasal allergic reaction, such as IgE production, eosinophil infiltration in nasal tissue, and allergen-induced symptoms. Histamines derived from mast cells and basophils play important roles in inducing allergic symptoms [35].

In fact, our results demonstrated that after OVA nasal irritation, Penh gradually increased and reached maximal values, while treatment with XG significantly reduced this increase compared to the standard care treatments. AR is a commonly caused by an allergic inflammation of the nasal mucosa mediated by IgE; therefore, it is characterized by prominent concentrations of allergen. For this reason, specific IgE antibodies, including the total and OVA-specific IgE levels, were evaluated, showing that mice stimulated with OVA had significantly elevated titers of serum total and OVA-specific IgE, while the treatment with XG significantly reduced IgE levels. Moreover, nasal histamine-responsiveness was significantly higher in OVA-sensitized mice compared to sham groups, indicated by the notable increase in histamine concentration during allergic rhinitis events. XG significantly reduced the clinical response at high logarithmic concentrations of histamine with similar efficacy to the standard of care treatments and especially to corticosteroids. Moreover, a main indicator of AR is the presence of eosinophils recruitment into the nasal mucosa; here we observed that the number of eosinophils in the nasal mucosa increased significantly in mice with OVA-induced AR, while XG significantly reduced nasal eosinophils infiltration.

In AR, inflammation of the nasal mucosa, together with an excessive differentiation of Th2 cells [36], provokes the secretion of several cytokines such as IL-4, IL-5, IL-10 and IL-13 [37].

In particular, IL-4 drives T-cell activation and differentiation into the Th2 subtype; at the same time, IL-4 and IL-13 stimulate IgE production in B cells and mucus provision in the airways [38].

Thus, the levels of the pro-inflammatory cytokines such as IL-4, IL-5, IL-12, IL-13, IL-33 and IL-25 were measured in BALF. We clearly observed that XG treatment significantly reduced the production of pro-inflammatory cytokines compared to cytokine levels detected in OVA stimulated mice.

These results confirm the central role of pro-inflammatory cytokines in the upregulation and activation of various cell populations involved in the allergic inflammation; however, our data reveal that XG was able to reduce several symptoms that drive allergic events.

Mucins are high–molecular-weight proteins and represent the main macromolecular factors of mucus [39]. The goblet cells of the superficial epithelium as well as the mucous cells of the submucosal glands represent the two main cell types most involved in the synthesis and secretion of mucins. Despite eight isoforms, includingMUC1, MUC2, MUC4, MUC5AC, MUC5B, MUC7, MUC8 and MUC13 [40], which are generally implicated in the human respiratory tract, only MUC5AC and MUC5B have been shown to be the major components of human airway secretion [41].

For this reason, we focused on the expression of the two secreted mucins and our results showed that AR mice display a significant increase in MUC5B and MUC5AC mRNA levels compared to sham mice. XG significantly reduces mucin expression with similar efficacy to the standard of care treatments indicating improved allergy response. The concomitant treatment with oral antihistamines significantly reduces MUC5B and MUC5AC mRNA levels with no significant differences in efficacy if compared to corticosteroid-treated mice.

The nasal epithelium is the first barrier of defense versus allergens, pollutants, and pathogens.

Since AR patients presented a defective epithelial barrier, we studied the responsible mediators in nasal secretions involved in AR. A disrupted epithelium allows microorganisms to penetrate, activating immune responses and leading to an allergic reaction; this supports the reason why defective and dysfunctional epithelial tight junctions are a common feature of AR [42].

Specifically, the mucosal barrier of the upper respiratory tract, which includes the nasal cavity, acts an essential role in host defense, thanks to the cooperation of innate immunity and tight junctions of epithelial cells. Indeed, tight junction proteins are expressed in both M cells and dendritic cells. Maintenance of intact and functional tight junctions is crucial to avoid inflammatory responses occurring in diseases such as allergic rhinitis [43]. Our results showed the crucial role of the tight junctions through ZO-1 and HDAC1. ZO-1 tight junction has an important role in the preservation of epithelial barrier function, while HDAC1 is a common feature of allergic rhinitis [7].

OVA-treated mice displayed a significant decrease in ZO-1 levels compared to the sham group, indicating a notable increase in barrier permeability. XG significantly decreases nasal barrier permeability with comparable efficacy to standard of care treatments. In the same way, OVA-treated mice displayed a significant increase in HDAC1 levels compared to the sham group, and the treatment with XG significantly decreases HDAC1 levels with comparable efficacy to standard of care treatments.

Therefore, the obtained results demonstrate that the treatment with the XG-based nasal spray provided a greater reduction of the inflammatory mediators that are involved in AR symptoms thanks to its ability to create a defensive biofilm on nasal epithelial cells and to prevent the contact of mucosal cells with antigens, thereby providing a greater overall relief of AR symptoms.

Our results represent a necessary step towards future clinical studies that could eliminate the problem of adverse effects observed with current therapies. In this way, other parameters should also be evaluated first in vivo and subsequently in the clinical setting. Such parameters include the study of pulmonary symptoms and function tests through forced expiration (FE)-related parameters.

In conclusion, we validated the non-pharmacological barrier abilities of XG, which could represent a promising, innovative approach to common drugs for the management of AR. Therefore, the use of a product containing compounds of natural origin could prevent and help manage allergic diseases, attenuating their symptomatology, reducing side effects of current treatments and thus improving the quality of life in AR patients.

## 4. Materials and Methods

### 4.1. Materials

Chemicals of the highest commercial grade available were used in this study. All stock solutions were prepared in non-pyrogenic saline (0.9% NaCl, Baxter, Milan, Italy). Xyloglucan-based spray was kindly provided by DEVINTEC Sagl (Lugano, Switzerland).

### 4.2. Animals

In this study, BALB/c male mice (6 to 8-week-old; 20–25 g, Envigo, Italy) were used. The animals had free access to water and food. The study was approved by the University of Messina Review Board. Animal care was in compliance with Italian regulations on protection of animals used for scientific purposes (D.M.116192), as well as with the EEC regulations (O.J. of E.C. L 358/1 12/18/1986).

### 4.3. Allergic Rhinitis Animal Model

Sensitization of the mice was carried out via an injection of 25 μg of ovalbumin (OVA; grade V; Sigma-Aldrich, St. Louis, MO, USA) and 2 mg of aluminum hydroxide on days 0, 7 and 14. The mice were then subjected to intranasal challenges with 100 μg of OVA for seven consecutive days from days 21 to 27. Differently, negative sham mice were injected, intraperitoneally and intranasally, with PBS until day 27. XG (20 μL of XG containing 0.02 μg per mouse) and saline (20 μL per mouse) were administered by intranasal instillation on days 21 to 27 (3 h before intranasal OVA challenge); no adverse effects due to the administration of XG were detected. For the other groups, we evaluated and compared the efficacy of a corticosteroid, antihistamine and mast-cell stabilizer treatment to XG-based spray, and also assessed the concomitant efficacy of antihistamines with the XG-based spray.

### 4.4. Experimental Groups

(1) Sham + vehicle (PBS): negative sham mice (N = 10);
(2) Sham + corticosteroid: mice were intranasally administered with spray budesonide (N = 10);
(3) Sham + antihistamine: mice were orally administered with oral tablet of cetirizine at the dose < 2mg/Kg for 7 successive days (N = 10);
(4) Sham + mast-cell stabilizer: mice were orally administered with oral tablet of cromoglycate at the dose < 82mg/Kg for 7 successive days (N = 10);
(5) Sham + XG: mice were intranasally administered with XG, for 3 successive days on 3 consecutive weeks (N = 10);
(6) Sham + XG + antihistamine: mice were administered with XG plus oral tablet of cetirizine (N = 10);
(7) AR + PBS: mice were intraperitoneally injected with OVA (10 μg OVA) on days 0, 7, and 14, and one weeks after OVA injection the mice received the instillation of a 10-μL droplet of OVA (N = 10);
(8) AR + XG: as AR, mice were intranasally administered with XG at the dose 0.02 μg/mouse on days 21 to 27 (3 h before intranasal OVA challenge) and sacrificed on day 28 (N = 10);
(9) AR + XG: as AR, mice were intranasally administered with XG at the dose 0.02 μg/mouse on days 21 to 27 (3 h before intranasal OVA challenge) and sacrificed on day 29 (duration of efficacy) (N = 10);
(10) AR + corticosteroid: as AR, mice were intranasally administered with intranasal spray of budesonide on days 21 to 27 (3 h before intranasal OVA challenge) (N = 10);
(11) AR + antihistamine: as AR, mice were orally administered with cetirizine at the dose < 2 mg/kg, on days 21 to 27 (3 h before intranasal OVA challenge) (N = 10);
(12) AR + mast-cell stabilizer: as AR, mice were orally administered with cromoglycate at the dose < 82 mg/kg, on days 21 to 27 (3 h before intranasal OVA challenge) (N = 10);
(13) AR + XG + antihistamine tablet: as AR, mice were intranasally administered with XG and orally administered with oral tablet of cetirizine on days 21 to 27 (N = 10).

To evaluate the duration of the mechanical barrier and the associated protective effects of XG could take at least 24 h. The animals were sacrificed at two different timepoints: at days 28 and 29. Mice were anesthetized intraperitoneally with ketamine and xylazine (2.6 and 0.16 mg/kg body weight, respectively), and then blood and bronchoalveolar lavage fluid (BALF) samples were collected from mice followed by centrifugation at 3000 rpm for 10 min. The plasma and BALF supernatant were stored at −80 °C for subsequent measurements. Moreover, the skull was sagittally bisected to remove septal and turbinate mucosa for histological examination.

### 4.5. Nasal Allergic Symptoms

Nasal allergic symptoms were evaluated by the number of sneezes and nose rubbing bouts counted over 15 min using a camcorder, as previously described by [35].

### 4.6. Assessment of Airway Reactivity

Airway reactivity was assessed through the methacholine-induced air-flow obstruction method, as previously described [44].

As reported by [45], we used the following equation: Penh = pause × (peak inspiratory boxflow/peak expiratory boxflow) in order to determine the enhanced pause (Penh) values for each methacholine dose.

### 4.7. Cytokine Quantification by ELISA and Cell Collection in Bronchoalveolar Lavage Fluid (BALF) and Nasal Mucosa Tissues

The BALF was performed as previously described by [46]. The cytokine quantifications of interleukin (IL)-4, IL-5, IL-12, IL-13, IL-33 and IL-25 were measured in the BALF and in the nasal mucosa of OVA-sensitized/challenged mice, using ELISA kits (Biolegend, San Diego, CA, USA and Invitrogen, Waltham, MA, USA) according to the manufacturer’s instructions.

### 4.8. Measurement of Total and OVA-Specific IgE

The levels of allergic mediators such as total and OVA-specific IgE were detected in the serum of OVA-sensitized/challenged mice. A total of 0.7 mL of blood was collected from each mouse via cardiac exsanguination. The total and OVA-specific IgE levels were determined using ELISA kits (Biolegend, San Diego, CA, USA, cat#RUO432404) in accordance with the manufacturer’s instructions.

### 4.9. Histology of Nasal Tissue

Nasal tissue was isolated from each mouse and the samples were stained with hematoxylin and eosin (H&E), as previously described [47].

### 4.10. Alcian Blue/PAS Staining

Alcian blue/PAS staining was employed to identify acid muco-substances and acetic mucins in different mucous secreting cells of the nasal mucosa, as previously described by [48].

### 4.11. RNA Isolation, cDNA Synthesis, and Real-Time Quantitative PCR Amplification

Total RNA was isolated from sinus mucosa tissue for RT-qPCR analysis using a Trizol Reagent Kit (Life Technologies, Monza, Italy). The first strand of cDNA was synthesized from 2.0 µg total RNA using a high capacity cDNA Archive kit (Applied Biosystems, Carlsbad, CA, USA). β-actin mRNA was used as an endogenous sham to allow for the relative quantification. RTqPCR was made for the evaluation of the following gene expression: MUC5AC (5′ CCAGCACCATCTCTACAACCC 3′ and 3′ GCAAAGCTCCTGTTTGCACTC 5′) and MUC5B (5′ GTGGCCTTGCTCATGGTGT 3′ and 3′ GGACGAAGGTGACATGCCT 5′) using Power Up Sybr Master Mix (Applied Byosystems, Carlsbad, CA, USA) and a QuantStudio 6 Flex Real-Time PCR System (Applied Biosystems, Carlsbad, CA, USA). The amplified PCR products were quantified by measuring the calculated cycle thresholds (CT) of target genes and β-actin mRNA. After normalization, the mean value of the normal sham target levels was chosen as the calibrator and the results were expressed according to the 2−∆∆Ct method as a fold change relative to normal shams.

### 4.12. Localization of Histone Deacetylases (HDACs) and Zonula Occludens (ZO)-1 by Immunohistochemistry Analysis

Immunohistochemical analysis was performed as already described [49].

Nasal tissue sections were incubated with the following primary antibodies: ZO-1 (Santa Cruz Biotechnology, Dallas, TX, USA; #sc-33725) and HDAC1 (Santa Cruz Biotechnology, Dallas, TX, USA; #sc-8410).

The histogram score is associated to the positive pixel intensity value achieved [50], and expressed as the percentage of the number of positive cells per high-power field.

### 4.13. Immunofluorescence Staining for ZO-1

An immunofluorescence procedure was performed as previously described [51].

Nasal tissue sections were incubated with ZO-1 primary antibody (Santa Cruz Biotechnology, Dallas, TX, USA; #sc-33725). Slices were examined and images were acquired at 40× magnification using a Leica DM2000 microscope (Leica, Milan, Italy).

### 4.14. Eosinophils Count by Sirius Red Staining

After the sacrifice of the animals, nasal mucosal tissues from the mice of each group were fixed in formaldehyde, embedded in paraffin, sectioned coronally into 7-μm slices and stained with Sirius Red in order to count eosinophils, as reported by [52]. Eosinophils number in the submucosal region of the nasal septum were quantified under a microscope in four fields at ×40 magnification in a blinded fashion.

### 4.15. Cell Line

A murine fibroblast L-929 cell line was purchased from ATCC (ATCC^®^ CCL1 ™; 10801 University Boulevard Manassas VA, 20110, USA). Cells were grown in culture flasks containing Minimum Essential Medium Eagle (Millipore Sigma, Burlington, MA, USA), supplemented with 10% fetal bovine serum (FBS, Millipore Sigma, Burlington, MA, USA) and 1% antibiotic-antimytotic solution (Millipore Sigma, Burlington, MA, USA). Cells were maintained at +37 °C in a humidified 5% CO_2_ atmosphere and monitored daily by using an inverted microscope. Subcultures were performed when 80% of confluence was observed.

### 4.16. MTT Assay

Cell viability of the L-929 cell line was assessed using a mitochondria-dependent dye for live cells (tetrazolium dye; MTT) to formazan. Cells were treated with XG at the concentration of 0.02 μg for 24 h. Cells were then incubated at 37 °C with MTT (0.2 mg/mL) for 1 h. The medium was removed and the cells lysed with dimethyl sulfoxide (DMSO) (100 µL), as previously described [53]. The extent of reduction in MTT to formazan was quantified by measurement of optical density at 550 nm with a microplate reader.

### 4.17. Statistical Analysis

Experimental data are expressed as mean ± standard error of the mean (SEM) of N observations, in which N represents the number of animals studied. The experiments are representative of at least three independent experiments. Data analysis was performed with One-Way and Two-Way ANOVA followed by a Tukey post-hoc test for multiple comparisons. Only a *p*-value less than 0.05 was considered significant.

## Data Availability

The datasets used and/or analysed during the current study are available from the corresponding author on reasonable request.
